# Sport and ageing: a systematic review of the determinants and trends of participation in sport for older adults

**DOI:** 10.1186/s12889-017-4970-8

**Published:** 2017-12-22

**Authors:** Claire R. Jenkin, Rochelle M. Eime, Hans Westerbeek, Grant O’Sullivan, Jannique G. Z. van Uffelen

**Affiliations:** 1 Institute of Sport, Exercise and Active Living (ISEAL),Victoria University, Melbourne, VIC 8001 Australia; 2 Facility of Health, Federation University, PO Box 663, Ballarat, VIC 3353 Australia; 3 Department of Movement Sciences, Physical Activity, Sports and Health Research Group, KU Leuven - University of Leuven, B-3000 Leuven, Belgium

**Keywords:** Older adults, Sport participation, Determinants, Trends

## Abstract

**Background:**

The global population is ageing. As ageing is often associated with a decline in health, there is a need to further develop preventative health measures. Physical activity can positively influence older adults’ (aged 50 years and older) health. Previous research on the relationship between physical activity and health for older adults has mainly focused on physical activity in general, and not specific types of exercise. Due to the social nature of sport, it may assist in improving physical, mental and social health for older adults. Sport, as a form of physical activity, has not been widely explored as a physical activity opportunity for older adults. This review concurrently explored two research questions: the determinants and the trends of sport participation for community dwelling older adults.

**Methods:**

Two parallel systematic searches of nine electronic databases were conducted in December 2015 for the two research questions. English language quantitative and qualitative studies that provided specific results for community dwelling older adults’ sport participation were included and a quality ratings assessment was undertaken.

**Results:**

There were 10,171 studies initially identified for the first research question and 1992 studies for the second research question. This culminated in 18 and 8 studies respectively that met the inclusion criteria. The most frequently mentioned determinants of participation were health and using sport to negotiate the ageing process. The most frequently mentioned trends of sport participation were the effect of historical sport participation on current participation, and sport participation across the lifespan. The main themes for both research questions had contrasting results, for example, participation in sport could improve health, but poor health was also a limitation of sport participation.

**Conclusions:**

This review demonstrates that older adults are a heterogeneous age group, and therefore require different strategies than other age groups to successfully participate in sport. It is recommended that the main findings from this review are incorporated into specific strategies to develop age appropriate sporting opportunities for older adults, so that sport can be presented as a viable physical activity option for this age group.

## Background

Populations throughout the world are ageing, and the amount of people aged over 65 years is shortly expected to outnumber children under five years old [1]. As people age, they are more likely to suffer from ill health, including chronic disease [2]. Physical inactivity is a significant contributor to the development of chronic diseases [3], therefore regular physical activity is important for older adults’ health and quality of life.

The health benefits of physical activity, specifically for older adults, have been comprehensively researched. For older adults, physical activity can be beneficial for physical [4], mental [5] and social [6] health. Besides the health benefits of regular participation, other aspects of participation among older adults have also been reviewed. Previous systematic reviews on physical activity in this population group have for example focused on: the physical health risks involved in participation [7], the differences between determinants of physical activity and exercise [8], whether older adults are meeting the recommended physical activity participation levels [9], the health benefits [10], strong social networks [11] and the effect of physical activity in alleviating depression in older adults [12]. Although the health benefits of generic physical activity have been extensively researched for older adults, there is little research on sport as a form of leisure-time physical activity for this population group. This limited research has largely focused on Masters/Senior Games sport participation [13–19] or on specific sports, such as bowls [20], golf [21], curling [22] or lifeball [23] rather than general community sport. As the determinants of participation may vary for different forms of exercise [24], specific research for older adults and general community sport participation is required. In this review, community sport is defined as “a human activity capable of achieving a result requiring physical exertion and/or physical skill which, by its nature and organisation, is competitive and is generally accepted as being a sport” [25].

Deriving benefits of physical activity can influence continuation of participation. In sport, there have been a number of systematic reviews examining the concept of sport being beneficial for health for younger age groups, [26–28] but not specifically for older adults, who may have different health outcomes. Therefore, with the expected declining health of an ageing population, it is important to investigate the determinants of initiating and continuing sport participation for older adults, to diversify the physical activity options available to this age group.

It is noteworthy that a recent narrative literature review [29] identified that whilst there can be numerous psycho-social benefits for some older adults who play sport, sport is a multi-faceted concept and as such, the socio-cultural contexts of older adults’ participation in sport needs to be considered. This current systematic review aims to build on the knowledge collated from this narrative review [29] in several ways. Firstly, to broaden the scope to review studies that also examine older adults’ participation in community-based sport clubs, as the majority of the literature in this narrative review related to large scale competitive events, like the Masters Games. Secondly, to look beyond solely subjective meaning found in participation to any influence that may determine sport involvement, such as demographics and also overall trends in participation.

Sport, as a type of leisure-time physical activity, is receiving increasing academic interest in ageing research [29–31]. Sport is often undertaken at community sport clubs in Australia [32, 33], and given the social nature of club-based sport, engaging older adults in sport may positively contribute to their physical, mental and social health. However, few older adults participate regularly in organised sport [34], therefore a review of the literature specific to older adults’ community sport participation is needed to identify potential determinants and trends of their participation.

This systematic review had two research questions: ‘What are the determinants of sport participation for community dwelling older adults?’ and ‘What are the trends of sport participation for community dwelling older adults?’ The decision to include both of these research areas in this review was based on the premise that determinants can influence patterns of sport participation either positively or negatively over the lifespan.

## Methods

### Search strategy

The two research questions led to two literature searches that were conducted in parallel, to identify research articles meeting the inclusion criteria for each of these questions.

Seven categories of determinants (biological, psychological, behavioural, physical, socio-cultural, socio-economic and policy) [35] that can influence participation or non-participation have been previously identified and were used in the inclusion criteria. The term ‘trends of participation’ was not specifically defined in the reviewed articles, so for the purpose of this review, trends were defined as participation levels or lifecourse participation. As Eime, Sawyer, Harvey, Casey, Westerbeek and Payne [36] state, the measure of sport participation trends is important for a range of sectors including sport, recreation and health. Other studies [37] have also investigated the determinants of participation in sport, but not for older adults.

The search terms for first research question were: [Sport* OR “sport* participation”] AND [Adult* OR “older adult*] AND [Determinant* OR reason* OR benefit* OR barrier* OR value]. For the second research question, the search terms were: Sport* OR “sport* participation” AND Adult* OR “older adult* AND Trend* OR lifecourse. The truncation symbol (*) was used to ensure all relevant uses of these search terms were included in the search.

Full database searches were conducted on 17th December 2015 across nine electronic databases: PubMed, Scopus, Cochrane, Cumulative Index to Nursing and Allied Health Literature (Cinahl), SPORTDiscus, AusSportMed, EBSCHOHost (including Health Business Elite, Health Source-Consumer Edition, Humanities International Complete, MEDLINE with full text, PsycARTICLES and PsycINFO, Informit), Psychology and Behavioural Sciences, and Health Collection. The searches were also limited to full English language peer reviewed articles.

### Selection criteria

In line with other recent research in older adults and sport [31], articles were included if there was specific data on sport participation in community dwelling adults aged 50 years or older (classified as older adults in this study). Sport participation was defined using the Australian Sports Commission’s definition [25]. Included studies also had to be restricted to older community-dwelling adults (for example, not in institutions such as hospitals). Furthermore, included studies were those that were empirically based (quantitative or qualitative) and published in peer reviewed journals. Articles were excluded if they were specific clinical or disability population studies and/or impairment or injury studies. If studies presented clear sport results in the abstract, they were included. However, as sport can be often defined in different ways and can be included as a type of physical activity or exercise, studies where it was unclear if there were specific sport results at the abstract stage were escalated to the full paper stage for review.

### Search process

**Fig. 1:**
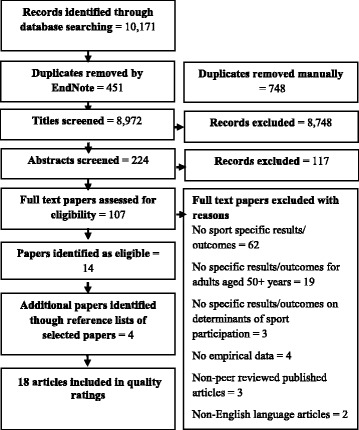
Search process What are the determinants of sport participation for community dwelling older adults?

**Fig. 2:**
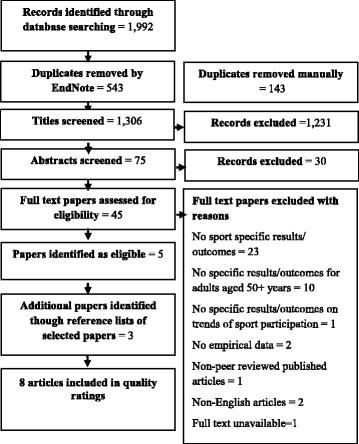
Search process: What are the trends of sport participation for community dwelling older adults?

The titles of studies were screened by one researcher. Abstracts were then screened by two researchers to ensure they met the inclusion criteria. The next stage involved two researchers screening the full text of articles to determine if they met the inclusion criteria. The reference lists of included full-text articles were then checked for additional relevant articles, which were also screened by two researchers for inclusion. Disagreements with regards to the inclusion of articles were discussed and resolved between these two researchers. See Figs. 1 and 2 for full details of this process.

### Study analysis

Once included, the articles were analysed thematically to report the main findings from each article that were relevant to the research question.

### Quality rating

The quality rating list developed by Kmet, Lee and Cook [38] was selected for the quality rating process, as it suitable for both quantitative and qualitative studies. This list has been cited extensively and used in numerous mixed methods systematic reviews [39–41].

The quality ratings system [38] involved 14 quality assessment items for quantitative studies and 10 items for qualitative studies. Items for quantitative studies covered appropriate research design and methodology, sufficiently appropriate data analysis and control for confounders. Items for qualitative studies covered a clear research question and context, use of theoretical framework, systematic data collection and analysis and consideration of reflexivity.

Response options (and score) for each quality rating item for both research designs were ‘yes’ (2), ‘partial’ (1), or ‘no’ (0) [38]. For the quantitative studies, a ‘not applicable’ (N/A) score was also used. There were three stages to calculate the overall score. Firstly, the total possible score was 28 minus (number of N/A’s ×2). Then the total score was (number of ‘yes’ ×2) plus (number of ‘partial’ ×1). The not applicable sections were excluded from the total score. Therefore the summary score was calculated by the total score divided by the total possible score. The final score for each qualitative study was calculated by: Total sum equalled (number of ‘yes’ ×2) plus (number of ‘partial’ ×1) divided by the total possible sum of 20. The not applicable option was not available for the qualitative score [38].

Two researchers independently undertook quality ratings in a pilot of four studies (two quantitative and two qualitative) for rigour. These researchers then discussed and resolved any discrepancies before independently undertaking quality ratings for the remaining studies. A Kappa score was calculated as an indication of agreement between reviewers and then any differences in ratings for the remaining studies were then discussed and resolved. Articles were classified out of a maximum score of 1.0, with strong articles categorised as >0.8, moderate (0.61–0.8) or weak (<0.6), based on the categories used by Henry, Kyle, Bhandari, Chisholm, Griffiths and Bundy [42]. Although Henry et al. [42] used an amended version of Kmet et al.’s [38] quality rating system, these categories were used as they most accurately reflected the authors’ opinion on the quality of the articles. The rating for each article is provided in Tables 1 (for search 1) and 3 (for search 2) in the Results section.

## Results

In total, 10,171 studies were initially identified for the first research question and 1992 studies for the second research question. There were 18 studies regarding the first research question and eight studies regarding the second research question that were included in the final review. These low inclusion rates were due to a number of studies either not clearly defining sport or not providing specific results for sport participation. Details of this process can be found in Figs. 1 and 2 respectively. The themes that emerged from the quantitative and qualitative studies are presented concurrently in a convergent style [43] throughout this review.

### Determinants of sport participation for community dwelling older adults

There were more qualitative studies (*n* = 10) than quantitative (*n* = 8) studies. The majority of the quantitative studies were cross-sectional (*n* = 5), with three longitudinal studies. Most of qualitative studies were interviews (*n* = 6), plus two studies utilising both ethnographic and interviews, one using photo elicitation and interviews and one study using photovoice and interview research methodology. The range of participants across included studies was six to 22,050. The majority of the studies were undertaken in Australia (*n* = 5) [13, 14, 20, 23, 44] and the USA (*n* = 5) [15–18, 21], with one study undertaken respectively in Canada [22], England [45], Germany [46], The Netherlands [47], New Zealand [19], Scotland [48], South Korea [49] and Sweden [50]. There were 13 studies that focused on the older adult age group, whilst three studies were non-specific to older adults, but reported age specific data. Details of the studies included in this section can be found in Table 1.

**Table 1:** Studies investigating the determinants of sport participation for community dwelling older adults

Ref & Year	Design*	Method	Sample (n)	Country	Age (yrs)	Cohort	Sex**	Aim (as reported by the author)	Sport and/or PA****	Sport***	Theory	Key finding(s)	Quality Score (out of 1.0)*****
[45] (2010)	Quant	Cross-sectional	2111	England	60–69	Community dwelling adults	M&F	Examine the reasons for the decline in physical activity	PA & sport	General sports		-Barriers included employment, lack of leisure time, physical limitations and poor health - There were few sport participation differences between employed and retired older adults, thus suggesting employment and lack of leisure time may not be a determinant of participation.	0.85
[44] (2011)	Quant	Cross-sectional	22,050	Australia	>65	Community dwelling adults	M&F	Characterise the types of leisure time physical activity in older Australians	PA & sport	General sports		- Older adults are more likely to engage with organised activity rather than unorganised activity, such as physical activity classes, rather than sport) - Most activities undertaken were aerobic types of activities, such as swimming, golf, cycling, racquet ball and rowing	1
[13] (2006)	Qual	Interviews	28	Australia	60–89	Masters athletes	M&F	Explore the motives and experiences of Australian Masters Games’ athletes	Sport	General sports	Post-structural	- Participants believed that their involvement in competitive sport prolonged their physical fitness, social health and psychological health. - Participants celebrated that their behaviour challenged age-appropriate norms and disassociated themselves from the aged stereotype - Resistance to the ageing body was associated with feelings of personal empowerment. However participants did not deny they were ageing, but wanted to enjoy playing sport for as long as they could	0.75
[23] (2009)	Quant	Longitudinal	284	Australia	40–96	Lifeball members/ex-members	M&F	Describe and examine the demographic and health related characteristics of Lifeball players and how these affect continuation in the sport	Sport	Lifeball******		Lifeball appealed to those who were already active, however poor health was the main reason for discontinuing playing Lifeball. Participants who had continued to play Lifeball 12 months after starting were more likely to report higher perceived socialisation benefits, but the quantitative data did not show any changes to level of physical activity, self-reported health status and quality of life	0.8
[50] (2012)	Qual	Interviews	22	Sweden	66–90	Active sports people	M&F	How sports can affect old adults’ processes of sense-making about old age	Sport	General sports	Grounded theory	- Participants used sport to maintain their ‘look age’, that is to maintain their weight, as a way to control the ageing process - Participants used sport as a way of evaluating and understanding old age, that is understanding how their physical capabilities were decreasing through participating in sport. Known as ‘capability age’. - Men measured capability age more quantitatively than women (through results of competition), and perceived ageing as a negative concept - Women accepted their physical decline but saw ageing as a more positive process, where they could feel empowered and would become inspirational to other women	0.75
[19] (2001)	Qual	Interviews	15	New Zealand	71–78	Masters’ Games participants	M&F	Examine the beliefs about the role and meaning of physical activity in later life	PA & sport	General sports		- An appropriate level of competition and fairness was deemed important in order to value and enjoy involvement - Whilst participants dropped out of sport within a few years of leaving school, they started played again, either informally or in organised competition, in their mid-50s or early 60s - Participants largely participated in team sports in their youth, but now participated in individual sports	0.5
[15] (2012)	Quant	Cross-sectional	408	USA	55–94	Senior Games’ participants	M&F	Describe the behaviours, importance of the reasons for participation and perceived outcomes associated with the North Carolina Senior Games	PA & sport	General sports		- Participating in the North Carolina Senior Games made a contribution to participants’ physical and social engagement, for example being physically active and socially interacting with their peers - Competition was important to participants but not as important as social reasons - As the Games was a structured year round programme, this enabled participants to be more active throughout the year. Also less educated participants (high school or lower) saw the social determinants of participation as more important than higher educated participants	0.9
[16] (2013)	Qual	Interviews	10	USA	52–71	Senior Games’ participants	M&F	Examine the experience of older adults participating in serious leisure to determine how this experience contributes to successful ageing	Sport	General sports	Serious leisure perspective	- Participants expressed the need to persevere through injury and illness, as they expected positive outcomes, such as training success or to ensure financial stability (through winning races) - Benefits of participation reported included physical and social benefits, such as social networking/developing friendships, physical fitness, enhanced self-image and fun, from their participation. - Participants have developed a specialised knowledge base of how to play a sport and this previous investment encourages them to continue participating as they age. - Participants reinforced their social identities through their sport participation	0.65
[20] (2005)	Qual	Ethnography & interviews	18	Australia	64–88	Bowls participants	F	Identify the objective career of lawn bowlers and the subjective interpretations the participants assign to the sport	Sport	Bowls	Serious leisure perspective	-Women can engage with a sport via various pathways, such as friends, family or life circumstances and for different reasons, therefore previous history is not always the main determinant - Some women thrived on the competition, whereas other participants enjoyed informal participation	0.8
[48] (2001)	Quant	Longitudinal	1710	Scotland	39–60	Community dwelling adults	M&F	Examine physical activity participation data for early and late middle age in the West of Scotland	PA & sport	General sports		Individual sports are undertaken more by men than women in late middle age and more differentiation by socio-economic status is seen in late middle age than early middle age	0.55
[17] (2014)	Qual	Photo elicitation & interviews	6	USA	56–70	North Carolina Senior Games’ participants	M&F	Use photo elicitation to examine the meanings associated with physical activity participation	PA & sport	General sports	Grounded theory	- Participants indicated that they were resisting the stereotypes of ageing imposed upon them by society and were defining what successful ageing meant - Participating in a mega event provided an opportunity to develop a sense of collective community through competition and friendship. - Participants distinguished themselves from other older adults through competition. - Participants used sport as a mechanism to transform their identity from ageing older adults to competitive athletes	0.85
[49] (2014)	Qual	Interviews	10	South Korea	66–83	Sport club members	M&F	Examine the benefits of serious involvement in leisure activities among older Korean sport club members	PA & sport	General sports	Serious leisure perspective	Serious involvement in sports club activities provided the participants with psychological, social and physical health benefits	0.65
[47] (2012)	Quant	Longitudinal	1460	The Netherlands	55+	Retired or widowed participants	M&F	Examine widowhood and retirement as determinants of moderate to vigorous physical activity and sports participation	PA & sport	General sports		No association between retirement or widowhood on sports participation, therefore not a determinant of participation	0.91
[22] (2011)	Qual	Photovoice & focus groups	15	Canada	12–72	Curling participants	F	Examine the influence of curling on the health of women in rural Canada	Sport	Curling		- Curling was vital to participants’ mental and physical health in winter - Playing curling can foster social connections and decrease social loneliness - Curling was seen as a way to support rural life. Participants volunteered and supported the club as an extension of supporting their community	0.8
[14] (2007)	Qual	Ethnography & interviews	110	Australia	55–94	Masters Games’ participants	M&F	How older adult Masters sport participants interpreted the concept of community	Sport	General sports	Grounded theory	- Participants developed feelings of belonging and membership with other participants through having a common interest in a particular sport - Being identified as a sports person whose very participation in sport was seen as an achievement, reinforced a feeling of relevance and life purpose. - Participants had shared desires to remain competitive, healthy and active in order to positively age - Older adults felt they had some influence and control in the sport they were playing by being able to “give back”, either through coaching or volunteering	0.9
[21] (2003)	Qual	Interviews	19	USA	67–87	Golf participants	M&F	Investigate the premise that serious leisure supports successful ageing	Sport	Golf	Serious leisure perspective	- Golf has different types of participants (core, moderate, social or therapeutic devotees) and therefore each group had different determinants to participation. - Participants enjoyed social health (social interaction and friends they had developed), Psych health (intellectual challenge, self-improvement, enjoyment, stress relief relaxation, pure fun) and PH (prevention of disability, as it kept them active and moving). - Golf was perceived to help some participants’ age well. It was a purposeful, meaningful activity and provided significant social relationships	0.65
[18] (1997)	Quant	Cross-sectional	246	USA	55+	Senior Olympics participants	M&F	Explore the influence of histories of competitive sports involvement, health beliefs, reasons for exercising and personality on physical activity participation	PA & sport	General sports	Health belief model	- Childhood and adolescent participation are not significant on Masters’ sport participation. However more than half of Masters’ participants still played sport in their 20s and 30s and others returned to sport during middle age rather than retirement. Suggests that some prior sport history is important but not all prior participation Competitors believed exercise was more important than non-exercisers, however they had more varied motivation to participate (improved health, in addition to socialisation and competency) than non-sports exercisers and non-exercisers	0.73
[46] (2010)	Quant	Cross-sectional	6569	Germany	50–67	Post-menopausal women	F	Examine the subject-related determinants of physical activity for post-menopausal women	PA & sport	General sports		- Sport participation was significantly associated with occupation (civil servants most popular), so job type can be a determinant for some participants - Also, later in life nulliparous women were less physically active than parous women, and women who had children at a younger age are less likely to participate in sport than older mothers, − Non-indigenous women was strongly associated with low sport participation	0.86

* Research design: Quant = quantitative research methods, Qual = qualitative methods. ** Sex: F = female, m = Male, M&F = both male and female. *** Sport: General sports = not one specific sport. A mix of different sports. **** Sport and/or PA: Sport = articles that only report on sport, PA & Sport = articles that report on both types of exercise but provide sport specific results. ***** Quality ratings: 08.-1 = good, 0.61–.079 = moderate, 0.0–0.6 = poor. ****** Lifeball is a team sport that is particularly suitable for older adults. It is a light intensity game that involves walking, passing and throwing a medium sized ball

The quality ratings ranged from 0.55 to 1. Most articles were rated as strong articles (*n* = 10), with six as moderate and two as weak. The inter-rater reliability Kappa score was 0.66 (*p* < 0.001) 95% CI (0.38, 0.94), which is classified as a substantial agreement [51] between the two reviewers. Seven main themes emerged from these 18 articles, as did several sub-themes, as shown below. These themes are presented in the order of most to least frequently mentioned in the included articles with an overview provided in Table 2.

**Table 2:** Summary of themes for the determinants of sport participation for community dwelling older adults

Theme	Sub-theme	Study
Health determinants	Health as a positive outcome of sport participation	13,15,16,21,22,49
	Health as a limitation of sport participation	16,23,45
Negotiating the ageing process through sport	Positive ageing discourse	13,14,16,17,21,50
	Negotiating the negative stereotypes of ageing	13,17
Social/community connection	Using sport to create/maintain a community	14,17,22
	Using sport to foster social connections	14,22
Influence of prior sports history on current sport participation	Positive influence of prior sports history	16,18,23
	Prior sports history may not always be important for current participation	20,21
Socio-demographic determinants	Employment/retirement	45,46,47
	Ethnic background	46
	Gender	50
	Occupation	46
	Parity	46
	Socio-economic status	48
	Marital status	46,47
Competition	To value sport	15, 19,20
	To distinguish participating from non-participating older adults	17
	Ensure financial stability	16
Sport type	Preference for individual sports	19,48
	Preference for organised activity	44

### Health determinants

Health was the most frequently reported determinant for older adults’ participation in sport, and there were two contrasting sub-themes: health as a positive outcome of sport participation; and physical health as a limitation to participation in sport.

There were six studies (one quantitative and five qualitative) which found that improved health was a positive outcome of sport participation. These articles reported that participants in Masters/Senior Games sport competitions [13, 15] and also in community sport clubs [16, 21, 22, 49] felt that participating in sport had assisted their physical [13, 15, 16, 21, 22, 49], mental or psychological [13, 16, 21, 22, 49] and/or social [13, 15, 16, 21, 49] health. However, there were three studies (two quantitative and one qualitative) that reported that poor physical health limited the ability of older adults to participate in sport [16, 23, 45].

### Negotiating the ageing process through sport

Negotiating the ageing process through sport was the next most frequently mentioned theme, which was highlighted in the qualitative studies only. This theme also had two sub-themes: positive ageing discourse; and negotiating negative stereotypes of ageing.

Developing a positive ageing discourse through sport was discussed by participants across many different studies [13, 14, 16, 17, 21, 50]. For example, older adults reinforced their social identity through participating in sport [16], used sport to differentiate themselves from non-active older adults [17] or used sport as a mechanism to transform their identity from an ageing older adult to a competitive athlete [17].

Two studies reported that participants used sport to negotiate the negative stereotypes of ageing [13, 17]. In one study, participants celebrated that their behaviour challenged age-appropriate norms and disassociated themselves from the aged stereotype [13]. Furthermore, several studies reported that sport was a purposeful and meaningful activity for older adults [14, 21, 50], whilst one study suggested that although their participants did not deny they were ageing, sport enabled them to resist their ageing body, which empowered them to enjoy playing sport for as long as they physically could [13].

### Social/community connection

Using sport as a tool to develop a social/community connection for older adults was a common theme amongst three of the qualitative studies, with two sub-themes emerging: using sport for the development and maintenance of community engagement for older adults; and using sport to foster social connections.

Two studies reported participant discussions of volunteering through sport or ‘giving back’ to the community. For example, one study suggested that supporting local sport clubs was seen as a way for older adults to support rural life/their local community [22], whilst another study reported that volunteering or coaching enabled participants to feel they had some influence or purpose in their sport [14]. For others, participating in a mega event provided an opportunity to develop a sense of collective community through competition and friendship [17].

The concept that sport can be used to foster social connections was discussed in two articles, one at a community sport club and one at a large sporting event. A study conducted in rural Canada suggested that playing curling could foster social connections and decrease social loneliness [22], whilst a study in Australia proposed that participants developed feelings of belonging and membership with other participants through having a common interest in a particular sport [14].

### Influence of prior sport history on current sport participation

The influence of prior sport history emerged in five articles (two quantitative and three qualitative), with contrasting sub-themes: positive influence of past sport history; and that prior sport history may not always be important for current participation.

Three studies reported that prior sport history had a positive effect on current older adult sport participation [16, 18, 23]. For example, these positive influences could be having prior competency, skills and knowledge to participate in sport [16, 18], or that sport was attractive to those already active [23]. Specifically for Masters sport participation, prior sport participation was important for some but not all participants [18]. Conversely, two qualitative studies suggested that prior sport history may not always be important for current participation, as different determinants influenced their participation [20, 21].

### Socio-demographic determinants

There were seven sub-themed socio-demographic determinants for sport participation that emerged from the quantitative (*n* = 6) and qualitative (*n* = 1) studies: employment and retirement, occupation, socio-economic status, ethnicity, gender, parity and marital status.

The influence of employment and retirement on sport participation was discussed in three studies [45–47]. One study found that retirement was not an important determinant of participation [47], whilst another study reported that employment was a perceived barrier [45]. A study from Germany reported that sport participation was significantly associated with occupation [46], with civil servants more likely to be engaged in sports compared to other types of workers, and another study suggested that a more favourable socio-economic status was a positive determinant in late middle age participation [48].

There was limited research that reported the influence of gender, ethnicity and parity on older adult sport participation. These studies reported that the ethnic background of participants was an important factor in one study. Being a non-indigenous female in Germany was strongly associated with low sport participation compared with indigenous females [46]. Gender differences in participation also emerged as a determinant from the one qualitative study. It was reported that men used their sport participation to measure their capability age, essentially assessing how their physical capabilities were decreasing, more quantitatively than women, largely through results in competition. Whilst the older women in this study accepted their physical decline, they saw ageing as a more positive process, where they could feel empowered and would become inspirational to other older women by participating in sport [50]. Parity was another socio-demographic sub-theme to emerge, with one study suggesting that women who had children at a younger age were less likely to participate in sport than older mothers [46].

Two studies assessed marriage, with one study suggesting that there was no association between marital status and sport participation [46], whilst another study showed no association between widowhood and sport participation [47].

### Competition

The importance, and enjoyment, of competition was reported in five studies (four qualitative and one quantitative) [15–17, 19, 20]. The role of competition was found to enable older adults to enjoy and value sport participation [19] or also to distinguish participants from older adults who did not play competitive sport [17].

### Type of sport structure

This theme included two sub-themes: preference for individual sports and preference for organised activity. Two studies (one quantitative and one qualitative) suggested that specifically middle aged men [48] and older adults in general [19], preferred participation in individual rather than team sports, whilst another quantitative study proposed that the ‘older old’ were more likely to engage with organised activity rather than unorganised activity [44].

## Trends in sport participation for community dwelling older adults

Relating to trends in sport participation, the majority of studies were quantitative (7), with one qualitative study. Most quantitative studies were cross-sectional (*n* = 5), one utilised both cross-sectional and longitudinal methods, and one was a cohort cross sectional study. The single qualitative study was an individual case study. A number of included studies did not provide overall sample size [52–55], however the number of participants across the quantitative studies that did report overall sample sizes ranged from 439 to 4199. The majority of the studies were located in Germany (*n* = 2) [52, 56], and Belgium (*n* = 2), [53, 54] with other studies undertaken in [54, 55] Japan (*n* = 1) [57], The Netherlands (*n* = 1) [58], Spain (*n* = 1) [53], and the USA (*n* = 1) [59] respectively. Four studies were non-specific to older adults, but reported age specific data, whilst four studies focused solely on the older adult age group. Details of the eight studies examining trends in sport participation for older adults can be found in Table 3.

**Table 3:** Studies investigating the trends of sport participation for community dwelling older adults

Ref & Year	Design*	Method	Sample (n)	Country	Age (yrs)	Cohort	Sex**	Aim	Sport and/or PA****	Sport***	Theory	Key finding(s)	Score (out of 1.0)*****
[52] (2009)	Quant	Cross & Long	3012–31,915	Germany	16- > 64	Community dwelling adults?	M&F	Determine whether the traditional assumption of decreasing sports activity with increasing age is still appropriate	PA & sport	General sport activities		In cross sectional analyses, sport participation was lower in older age groups. However longitudinally participation decreases with age for men but not women	0.65
[58] (2013)	Quant	Cross	4199	The Netherlands	58–67	Retired older adults	M&F	Investigate the trend in sport participation among retirees between 1983 and 2007	PA & sport	General sports		Successive cohorts of retirees are increasingly likely to participate in sport compared to pre-1983 Level of education can positively affect sport participation in later life, however physical limitations can negatively affect sport participation in later life	0.9
[56] (2011)	Quant	Cross	1739	Germany	50+	Community dwelling adults	M&F	Describe sport participation across the life course, and to what extent people’s previous experience of sport influences the decision to enter, return or exit participation in sport	Sport	General sports	Lifecourse approach	The longer a participant engaged in sport, the less likely drop out occurred. If participants started sport before the age of 30, they were more likely to leave it in the following 10–15 years than those who began playing sport at an older age. Women are more likely to start playing sport in later life than men. The timing of the introduction of sport policy seems to have a stronger effect on participation for those who had reached no older than middle adulthood at the time it was introduced than prior history in sport	0.77
[57] (1994)	Quant	Cross	439	Japan	60+	Masters & non-Masters participants	M&F	Compare two very different elderly populations to examine the diversity or heterogeneity in sport participation over time	PA & sport	General sports	Continuity	Masters sport participants played sport over the lifespan and reflect concepts in continuity theory. However, lifelong sport participation for non-Masters participants is more varied than Masters participants	0.59
[59] (1999)	Qual	Case study	1	USA	68	Retired older adults	M	Explore through narrative inquiry the events that characterised the life story of a senior-aged competitive sport participation	Sport	Baseball & Tennis	Continuity	Participant intimates that he was engaged in sport as a child and has continued his engagement in sports, albeit different sports dependent on life stage, into older age	0.85
[53] (2012)	Quant	Cross	5160–22,255	Spain	15–74	Community dwelling adults	M&F	Assess the trend in prevalence of Spanish adults who engaged in sports activities from 2000 to 2010	PA & sport	General sports		Sport participation from 2000 to 2010 showed a decrease in participation for older adults	0.7
[54] (2005)	Quant	Cross	8624–38,376	Belgium	19–77	Community dwelling adults	M&F	Examine stratification patterns with regard to different modes of sport participation	PA & sport	General sports		Sport participation decreases with age. A change in sport policy has ensured active involvement in sport is now socially acceptable for whole population	0.6
[55] (2011)	Quant	Cohort cross	5851–51,808	Belgium	19–90	Community dwelling adults	M&F	Analyse social stratification patterns in adults’ sports participation	PA & sport	General sports in sport clubs		Influence of adolescent sport participation decreases over time. Older adults less likely to participate in club-organised sport than younger people	0.65

* Research design: quant = quantitative research methods, qual = qualitative methods. ** Sex: F = female, M = male, M&F = both male and female. *** General sports = not one specific sport. A mix of different sports. **** Sport and/or PA: Sport = articles that only report on sport, PA & Sport = articles that report on both types of exercise but provide sport specific results. ***** Quality ratings: 08.-1 = good, 0.61–.079 = moderate, 0.0–0.6 = poor

Quality ratings for these studies ranged from 0.59 to 0.90. Most studies were rated as moderate (*n* = 4), with two strong studies and two weak studies. The inter-rater reliability Kappa score was 0.46 (*p* < 0.004) 95% CI (0.030, 0.88), which is classified as a moderate agreement between the reviewers [51]. Three main themes and several subthemes emerged from the study review, as detailed in Table 4 below.

**Table 4:** Summary of themes for the trends of sport participation for community dwelling older adults

Theme	Sub-theme	Study
Effect of historical sport participation on trends	Continuity theory	57,59
	Engagement in sport	55,56,57
Sport participation across the lifespan	Sport participation decreases with age	52,53,54
	Sport participation does not always decrease with age	52,58
Demographic impacts on sport trends	Policy/programming	54,56
	Education	58
	Health	58
	Gender	56
	Type of sport	55

### Effect of historical sport participation on trends

There were four studies (three quantitative and one qualitative) that reported the effect of previous sport participation, such as in childhood, adolescence or early adulthood, on current participation [55–57, 59]. Two sub-themes emerged from this theme: Continuity theory and engagement in sport.

Continuity theory, which states that adults use strategies linked to their past experiences (for example, sport participation) to adapt to the ageing process [60], was a key theme found in several studies. Two studies found the role of participation in sport specifically, or physical activity more generally, as a child or adolescent influenced participation in sport as an older adult [57, 59].

Trends in sport engagement were found to be more diverse in three other quantitative studies. For example, participation for some older adults was more varied [57]. One study found that the influence of adolescent participation decreased over time [55], whilst another study suggested that those who started playing sport at an older age were less likely to drop out than participants who had started playing at a younger age, for example, during adolescence [56].

### Sport participation across the lifespan

Four quantitative studies examined whether sport participation decreased with age, finding contrasting results. In the cross-sectional studies, it was reported that sport participation was lower in people with higher age [52–54]. However, one longitudinal study found that whilst sport participation decreased with age for men, it did not necessarily decrease for women [52]. Conversely, one study found that successive cohorts of retirees are increasingly likely to participate in sport [58].

### Demographic impacts on sport trends

There were five quantitative studies that observed demographic impacts on sport trends, with sub-themes of policy/programming, education, health, gender and type of sport structure. Two studies focused on sport policy, with both studies suggesting that the ‘Sport for All’ policies established in the 1960s, which have promoted sport participation for health and social benefits, had a strong effect on sport participation levels [54, 56]. Other studies stated that level of education can positively affect sport participation in later life [58], physical limitations can negatively affect sport participation in later life [58] and older adults were less likely to participate in club-organised sport than younger people [55]. One study also suggested that women were more likely to start playing sport in later life than men [56].

## Discussion

This is the first systematic literature review to explore the determinants and trends regarding older adults’ sport participation. Given the increasing ageing population of Western nations and the anticipated associated decline in health, it is important to investigate the potential of diverse forms of physical activity to enable age appropriate opportunities for older adults to undertake enjoyable exercise as they age.

### Determinants of sport participation for community dwelling older adults

There was a variety of common factors associated with sport participation in older adults, including health determinants, negotiating the ageing process through sport, social/community connection, the influence of prior sport history on current sport participation, socio-demographic determinants, competition and sport type. The most frequently reported themes were health determinants and negotiating the ageing process through sport.

It is not surprising that health was the most frequently mentioned determinant. An increasing ageing population is likely to increase the risk of chronic disease for the individual, and is also predicted to result in higher public health expenditure [1, 61], thus becoming a priority action area for governments. This review has shown that sport can provide positive health benefits for older adults, but also that sport participation may be more difficult for older adults as they age, because ageing is typically associated with a decline in health. This is especially prevalent for certain sports, such as contact or physically demanding sports. This concept is similarly reflected in studies on generic physical activity and older adults [62–64]. In this review, it is interesting to note that health as a motivation to exercise was mostly supported by data of older adults who played sport either in a community sport club or at Masters/Senior Games competitions [13, 15, 16, 21, 22, 49].

Poor health as a limitation to participation is a unique determinant for this age group and needs to be taken into consideration. As such, to enable older adults to derive the health benefits sport can provide, age appropriate playing opportunities are required, especially in more exertive sports, to accommodate for older adults who may have age related reduced physical capabilities. However, previous research has highlighted that many sports do not prioritise older adults specifically [31], but there is an opportunity for sport to be promoted as a novel intervention for health promotion in older adults.

Similarly to health determinants, this study also highlights another unique concept for older adults; that is, the role of negotiating the ageing process through sport. Studies in this review suggest older adults use sport to distance themselves from the older adult societal stereotype and/or reinforced their social identity through sport. For example, some older adults used sport to differentiate themselves from non-active older adults or used sport as a mechanism to transform their identity from an ageing older adult to a competitive athlete [17]. This concept has been previously identified by Dionigi’s [29] narrative literature review on the psychosocial and sociological issues of sport and ageing. The relevant studies on this theme in both Dionigi’s [29] review and this systematic review were all qualitative, as the data was mainly explorative and this concept may be difficult to measure quantitatively. However, it could be interesting to further develop this and to explore if quantitative evidence can reinforce this concept on a wider scale. Also, most of the current data on this determinant were for Masters or Senior Games sport participation, meaning older adults playing competitive sport at large scale events, but not in community level sport. Therefore further research should be conducted in community sport to investigate if similar concepts emerge in an informal and more social sport setting. This could enable sporting organisations to further promote sport as an attractive option for older adults to be physically active.

There were only a small number of studies examining socio-demographic factors associated with participation. Previously it has been found that socio-demographic determinants, such as gender, socio-economic status and ethnicity, are major factors associated with sport participation in other age groups, such as children/adolescents [65] and adults [66]. Conversely, from this review, it would appear that health as a determinant of older adult participation has more traction because it has public health and economic value. However, if organisations want to increase the proportion of older adults’ participating in sport to improve their health, then more knowledge on other potential determinants is needed, starting with socio-demographic determinants such as gender and education. In addition, as older adults have different life priorities and different impacts on their time than younger adults, it should be examined which age specific socio-demographic factors are associated with participation. For example, the unique impact of work/retirement as a determinant was only researched in three studies. More research on this is needed to further understand its potential influence on older adult sport participation and to be able to use this to identify subgroups of older people who may be interested in participating in sport.

### Trends of sport participation for community dwelling older adults

Overall there were fewer studies that investigated participation trends than participation determinants. From those participation trend studies, three main themes emerged. These related to the effect of historical sport participation on trends, sport participation across the lifespan and demographic impacts on trends.

This review found that in general, participation in sport declines with age. This is a similar finding to some generic physical activity research [67, 68]. Furthermore, historical/past participation in sport was found to be a determinant of current participation, which is also in line with determinants reported in previous physical activity [69] and sport research [70]. However, some themes from this review suggested that participation did not always decrease with age, which is also reflected in some adult leisure-time physical activity studies. These studies have reported that leisure-time physical activity for both men and women was actually increasing over time [71, 72]. This demonstrates the heterogeneity of this age group and can encourage sporting organisations to promote sport participation to older adults, regardless of their prior sport history [19, 73].

### Correlation between the research questions

The two research questions were amalgamated in this one systematic review because determinants can influence patterns of sport participation either positively or negatively over the lifespan. Additionally, some of the reviewed trend studies sought to identify factors, for example, demographics, that influenced patterns or trends over time. Overall, there were a number of findings that linked the two research areas.

Conflicting data on the influence of prior sport participation emerged for both research questions. As proposed earlier in this discussion, this suggests that older adults are a diverse age group who can be attracted to sport for the first time at an older age, if appropriate participation opportunities are provided.

Socio-demographic factors also emerged in both research areas, though the number of studies was relatively small. As these influences, such as gender, socio-economic status, physical environment and family background, have been shown to be influential for other age groups [74–76], it is recommended that more research on these influences is undertaken to further investigate these findings and understand their importance (or lack of) for this age group. Furthermore, the influence of retirement, a unique theme for older members of this age group, was inconclusive. This influence reported conflicting results between the one determinant study and one trends study that reported on this, thus further research is recommended for this theme to resolve this conflict. It is recommended that sporting organisations consider these socio-demographic influences when developing appropriate playing opportunities for this age group.

Health was a well-researched area for determinants, but there was only one trends study that included health. This trends study focused on how physical limitations can negatively affect sport participation in later life, which corresponded with some of reviewed determinant studies. These results reflect that older adults, as a group, are more likely to experience various health conditions that may affect their participation in some sports, such as those requiring high exertion or physical contact. There are unique determinants across different times within the lifespan, for example a study on sport participation for adolescents [77] have shown that having fun with friends and having role models were important determinants of sport participation. Whilst some of these determinants are the same for older adults, the results in this review suggest that appropriate opportunities should be developed in some sports for those with health limitations (such as adapted sport and/or social sport). This is in addition to age appropriate opportunities for those without debilitating health conditions, to also enable these older adults to continue playing either competitive or social sport with their peers.

### Methodological recommendations

According to Kmet et al.’s guidelines [38], the majority of the studies across both research areas were identified as high quality, however this research area could benefit from a number of methodological improvements. Qualitative research needs to better acknowledge the importance and potential impact of reflexivity, and quantitative research would benefit from more longitudinal designs to expand on the current, largely cross sectional, findings.

Many of the studies included in this systematic review were on Masters/Senior Games sport. That is, a competitive form of sport, rather than community-based recreational forms such as club sport. In the interest of population health strategies, the role of sport in the general community for older adults is important to understand. Whilst there were some studies on community sport, these tended to be sport specific and focused on traditional older adults’ sport, such as golf and bowls. Community sporting organisations can benefit from understanding the trends and determinants of participation, to better cater specific products/programmes to the needs of older adults. Community sport in recent years has developed and implemented specific modified sport products for very young children, to meet their developmental needs [78]. Given the different potential health limitations of some older adults, partial modifications of sports for older adults is a potential strategy to encourage their increased participation, at least for sports with high exertion or physical contact.

### Strengths and limitations of this review

This review presented two research questions to provide context and enable direct comparison between two important influences for older adults’ sport participation. It also comprised of both qualitative and quantitative studies to ensure a breadth of research was included. However, it also had a number of limitations. Whilst the search strategy was comprehensive, it did not include grey literature or non-English language articles. Also, whilst the quality rating system used enabled both types of studies to be assessed, the assessment criteria for the quantitative studies were not as rigorous as other quantitative rating systems, which may have impacted the assessment scores.

## Conclusion

This study brought together a range of diverse research investigating the factors associated with sport participation in older adults. As the populations of Western nations continue to age, it is important to explore different ways that older adults can be physically active in their leisure-time. In conclusion, this review highlights that older adults do use sport to improve their health, but at the same time, due to decreased health in general with ageing, poor health can equally be a barrier to participation. It is recommended that sporting organisations use this review to understand the determinants and trends of older adult participation, by providing both social play and competitive appropriate opportunities. However, it is essential that organisations ensure that these opportunities also cater for older adults who may have potential health limitations, to ensure older adults who enjoy sport can continue to participate as they age.
